# Negative Pressure Wound Therapy in Pharyngocutaneous Fistula

**DOI:** 10.7759/cureus.60457

**Published:** 2024-05-16

**Authors:** Muhammad Fawwaz Meor Abdul Malik, Mawaddah Azman, Marina Mat Baki

**Affiliations:** 1 Otolaryngology - Head and Neck Surgery, Universiti Kebangsaan Malaysia Medical Center, Kuala Lumpur, MYS

**Keywords:** laryngectomy, laryngeal carcinoma, negative pressure wound therapy, vacuum-assisted closure, pharyngocutaneous fistula

## Abstract

Pharyngocutaneous fistula (PCF) is an abnormal connection between the pharynx and skin that can occur after laryngectomy surgery. It can have a significant negative impact on patient recovery, delaying wound healing, requiring prolonged nil-per-oral (NPO) status, and reducing quality of life. Traditionally, the management of PCF has relied on conservative measures or surgical intervention. However, negative pressure wound therapy (NPWT) offers a promising alternative approach. This case study involves three patients who underwent laryngectomy and developed postoperative PCF. All patients received NPWT with a modified suction catheter and low negative pressure (20-40 mmHg). With NPWT, all patients achieved complete wound closure, with healing times ranging from two weeks to six weeks. This suggests that NPWT may significantly accelerate PCF healing compared to traditional methods. However, maintaining an airtight dressing on the neck region can be challenging. This study highlights the potential of NPWT for faster PCF closure after laryngectomy. Further research is needed to optimize NPWT application techniques, explore the impact on long-term outcomes, and establish guidelines for broader clinical use.

## Introduction

Negative pressure wound therapy (NPWT), also known as vacuum-assisted closure (VAC) dressing, is a technique commonly used in the management of complex wounds. While it is primarily used in the treatment of chronic wounds and open surgical wounds especially in the region of the trunk and limbs, it can also be applied in specific cases of pharyngocutaneous fistula (PCF) [1]. PCF refers to an abnormal communication between the pharynx and the skin of the neck. It can occur as a complication following surgery, such as laryngectomy or pharyngectomy, or as a result of trauma, infection, or radiation therapy. PCF will severely affect patients as they are usually prohibited from oral intake, require long hospital stays, and occasionally require surgery for closure [2]. Herein, we report a series of cases where NPWT dressing was used in managing patients with PCF after the laryngectomy surgery.

## Case presentation

Case 1

**Figure 1:**
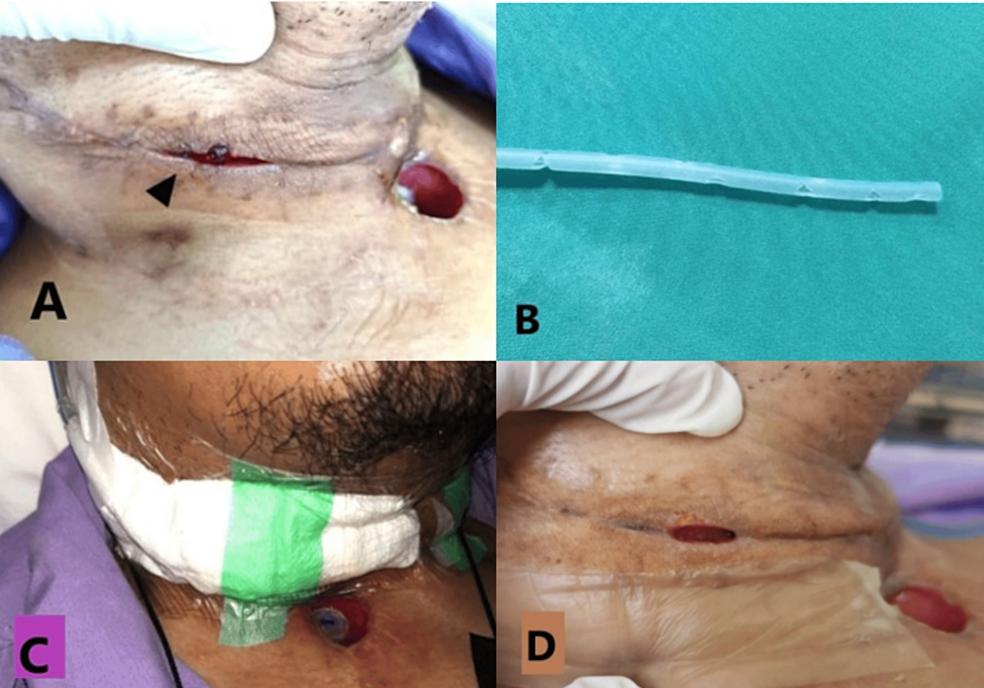
PCF at three weeks post-surgery (arrowhead) (A). Modified suction catheter with multiple fenestrae at the tip (B). NPWT was applied over the wound and connected to the wall suction unit (C). PCF at five weeks after surgery. Notice hydrocolloid dressing applied over the inferior edge of the wound (D). PCF, pharyngocutaneous fistula; NPWT, negative pressure wound therapy

A 38-year-old male underwent total laryngectomy, bilateral lateral selective neck dissection, hemithyroidectomy, and primary tracheo-esophageal puncture (TEP) with prosthesis insertion for laryngeal squamous cell carcinoma (T4aN0M0). Intraoperative and immediate postoperation was uneventful. He was nursed in the general ward for postoperative rehabilitation and tracheal stoma wound care and was discharged well on day 10 postoperatively with a nasogastric feeding tube. On day 19 after the surgery, he complained of right neck swelling with purulent discharge from a small area of wound gaping at the superior aspect of the stoma. The surgical scar was reopened at the right neck and 3 mL of hemopurulent discharge mixed with saliva was drained (Figure 1A). A barium swallow study showed a contrast leak at the region of the right hypopharynx. He was kept nil per oral afterward and NPWT was applied to the right neck wound.

NPWT was prepared using a modified suction catheter with few fenestrae at the tip (Figure 1B), sterile gauze, large transparent adhesive film, and sterile solution. The wound was cleaned with a hypochlorous acid solution and all the slough and debris were removed. The modified suction catheter is inserted into the wound making sure the fenestrae is inside the wound cavity. Gauze is then placed externally and the wound is covered with a large clear transparent dressing. The dressing was trimmed to fit the wound bed, ensuring it extends beyond the wound edges by a few centimeters (Figure 1C). The other tip of the tube was connected to the wall suction unit with a suction power of 40 mmHg. The dressing was done and reapplied every three days. Another barium swallow study was done on day 27 after surgery that showed a residual contrast leak at the right neck, which reduced in size comparatively. NPWT continued until the right neck wound healed at six weeks after the surgery (Figure 1D). Subsequently, he underwent adjuvant radiotherapy (RT) two weeks after completion of NPWT. Currently, the patient is on monthly follow-up with no evidence of recurrence at four months after completion of RT.

Case 2

**Figure 2:**
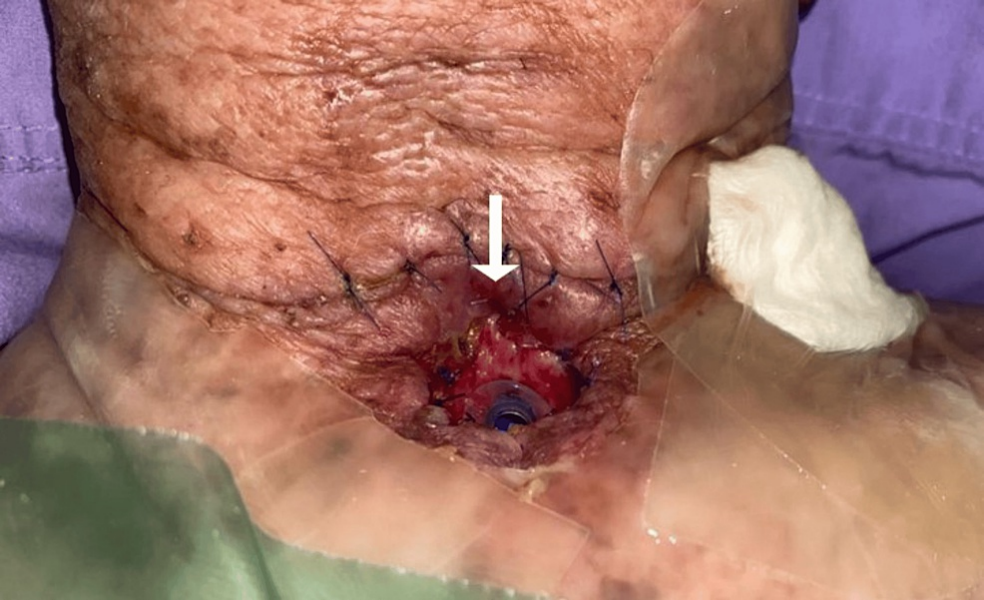
PCF at left neck on NPWT at two weeks after surgery. Notice the surrounding skin dressed with a thin hydrocolloid for protection and a healing fistulous opening superior to the stoma (arrow). PCF, pharyngocutaneous fistula; NPWT, negative pressure wound therapy

A 74-year-old male was diagnosed with recurrent glottic carcinoma rT3N0M0 after multiple excisions via transoral laser cordectomy. Subsequently, he underwent total laryngectomy, bilateral lateral selective neck dissection, total thyroidectomy, and primary TEP with prosthesis insertion. Intraoperatively was uneventful and he was nursed at the general ward postoperatively. However, on day 7 after surgery, he was noted to have saliva with pus discharge from the neck wound and at the superior edge of the tracheostoma. A corresponding left-sided neck swelling was seen as well (Figure 2). The surgical scar was intentionally reopened on the left side of the neck and treated conservatively with NPWT dressing. NPWT technique was similar to the first case and stopped once the wound improved with no deep cavity and discharge. Subsequently, daily ointment dressing was applied to the wound until completely healed. Botulinum toxin type A was injected over bilateral parotid glands and submandibular glands on day 11 after surgery. A total of 25 units of botulinum toxin type A were injected into each gland under ultrasound guidance to reduce saliva production.

He underwent a barium swallow study three weeks after surgery, which showed no anastomotic leak, and he was allowed oral intake. Currently, the patient is on surveillance follow-up monthly with no evidence of recurrence at six months after completion of RT.

Case 3

An 82-year-old male was diagnosed with recurrent glottic carcinoma rT3N0M0. Subsequently, he underwent total laryngectomy, bilateral lateral selective neck dissection, hemithyroidectomy, and primary tracheoesophageal puncture (TEP) with prosthesis insertion. Intraoperatively it was uneventful, and he was nursed at the general ward postoperatively. A barium swallow study done on day 10 after surgery showed no leak and he was allowed oral intake and subsequently discharged home.

**Figure 3:**
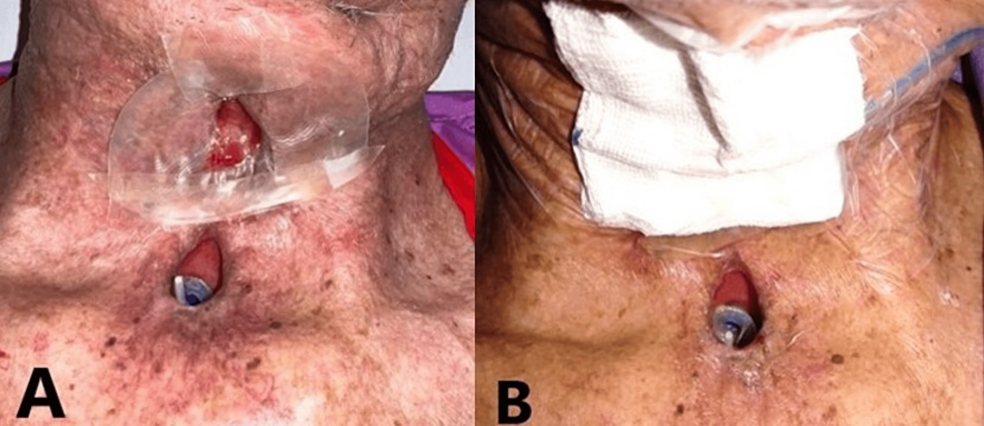
PCF at four weeks after incision and drainage. Notice the wound is clean and use of hydrocolloid dressing surrounding the wound to protect the edges of the wound (A). NPWT was performed and a transparent dressing cover on top of the gauze (B). PCF, pharyngocutaneous fistula; NPWT, negative pressure wound therapy

Three weeks after the surgery, he presented at another center with an extensive neck abscess where an incision and drainage were performed. Unfortunately, a generous neck incision was performed involving the stoma. Saliva and pus were evident from the wound bed, confirming the diagnosis of a PCF. A daily dressing with povidone-soaked ribbon gauze was done until the wound was clean, and then NPWT dressing was commenced at four weeks after surgery (Figure 3A, 3B). He showed good progress, and the wound completely healed at seven weeks after the surgery. A repeat barium swallow study was performed eight weeks after surgery, which showed no evidence of contrast leak or fistula. Currently, the patient is on monthly follow-up with no evidence of recurrence at one year after completion of RT.

## Discussion

Laryngeal carcinoma is one of the most common head and neck malignancies especially among populations with higher rates of alcohol consumption and smoking [3]. In cases of advanced laryngeal carcinoma, multimodality treatment options include surgery, RT, and chemotherapy. Total laryngectomy is the mainstay surgical treatment for advanced laryngeal carcinoma followed by adjuvant RT with/without chemotherapy. However, it carries significant morbidity with high complication rates reported as 40-92% [2]. One of the most common complications is PCF. It is characterized by the formation of an abnormal tract between the pharynx and the skin of the neck following total laryngectomy. The reported incidence of PCF ranges from 3% to 65% and most commonly occurs on days 7-11 after surgery [4,5]. There are a lot of risk factors that are associated with PCF such as previous RT, smoking, advanced age, tumor staging, and type of treatment either primary or salvage surgery [6]. PCF will delay patient recovery after major surgery and severely affect patients' quality of life as they are prohibited from oral intake and longer stay in the hospital [4]. Furthermore, a delay in PCF closure will further delay adjuvant RT. The recommended waiting time for adjuvant RT is six weeks, which delays the treatment and worsens the prognosis and local control of the disease [7].

NPWT, also known as VAC dressing, has been used as a treatment option for PCF. NPWT involves the application of a specialized dressing that uses negative pressure to help promote wound healing. The occlusive dressing is applied to the wound, and the negative pressure helps to remove excess fluid, reduce swelling, and promote new vascularization, thus, improving the growth of new tissue [4]. NPWT has been shown to be effective in promoting healing and reducing the size of PCF [1]. It is a standard treatment in complicated and intractable wounds over the trunk and limbs. However, in the head and neck region, it is not widely used due to difficulty in sealing the dressing due to numerous undulation on the skin surface, and also if the dressing is not airtight, salivary contamination can readily happen and worsen the infection [8]. Furthermore, exposed major vessels such as the carotid artery and internal jugular vein, especially after neck dissection and flap reconstruction surgery, are contraindications for applying NPWT [1]. There were reported articles in which the median time from the onset of fistula to restart oral intake for the conventional dressing in the management of PCF was 85 days; however, in NPWT dressing the mean duration was about 30 days [1]. Although it was not a direct comparison study, the reduction of duration was significant and, thus, improved patient prognosis with in-time adjuvant RT and reduced the cost of hospitalization. It is almost similar to our reported cases in which the duration of NPWT ranges from 12 days to three weeks.

There are also other advantages of NPWT in the management of postoperative PCF, which include better control of saliva leak. NPWT can minimize saliva and exudate outflow through the fistula by collapsing the fistula tract due to its negative pressure. Thus, it prevents contamination of the wound and reduces the risk of saliva-associated dermatitis [1]. NPWT also promotes wound healing by improving bacterial clearance, increasing blood flow, and stimulating granulation tissue growth [8]. As seen in our case, NPWT helps to direct saliva from PCF from soiling the wound connecting it with a controlled leak. Therefore, incisions need to be planned in non-critical areas. Although fistulous openings tend to involve the peristomal region, it is preferable to make an incision at the lateral neck where the skin flap is thick and, hence, would heal spontaneously with time.

There are a few conditions in which NPWT is contraindicated. It includes the use of NPWT on the exposed blood vessel, which can cause erosion of the vessel wall. Other than that, it is also contraindicated in a case of suspicious tumor clearance from the wound bed and infected wound with the presence of frank pus and necrotic tissue. Khoo et al. also suggested not to use NPWT on the fistula near the peristomal region due to the risk of aspiration [8].

Many articles describe how and what type of NPWT was performed. Some used a VAC system (KCI, Dallas, TX), RENASYS (Smith and Nephew Healthcare, Hull, UK), WOUND assist (HNE medical, Limonest France), continuous low-pressure suction unit (MERA Sacuum; Senko Medical Instrument Manufacturing Co., Tokyo, Japan) or NPWT using a wall suction apparatus [1,9]. The negative pressure was kept between 80 and 125 mmHg [1,4,10]. In our cases, we used a modified tube with multiple fenestrae at the tip, which was covered with a sponge or gauze and connected to wall suction, and negative pressure was kept low between 20 and 40 mmHg and changed every three days. Occlusive dressing can be achieved with the use of hydrocolloid dressing on the skin. It can help protect the surrounding skin and improve adhesiveness. Opsite Transparent Waterproof Films (Smith+Nephew, Baar, Switzerland) was used to seal the system [4].

Botulinum toxin type A is a selective anti-cholinergic drug that can reduce the production of saliva transiently. It will block the release of acetylcholine at cholinergic nerve terminals, which will result in reduced salivation. It has been reported that botulinum toxin was used to treat Frey syndrome. This syndrome occurs due to aberrant reinnervation from secretory nerve fibers of the parotid gland to the sweat glands. This botulinum toxin injection inhibits the release of acetylcholine and blocks the misdirected secretory nerve fiber [11,12].

In general, patients with postoperative PCF are prohibited from oral intake. In all reported cases above, patients were kept nil per oral and were fed by nasogastric tube or gastrostomy/jejunostomy tube until the PCF wound healed. However, there is a reported article in which they start to allow patients to feed by oral route once the fistula shrunk sufficiently and occlusive dressing can be maintained [1,2].

## Conclusions

NPWT is one of the good treatment options for PCF. It can help fasten the wound healing and further reduce the morbidity and cost. One of the main challenges of VAC application is to maintain occlusive dressing. The application of flexible hydrocolloid dressing may be useful to achieve air-tight dressing. It can avoid trauma over the PCF surrounding skin and also improve the adhesiveness of the dressing.

## References

[1] Inatomi Y, Kadota H, Yoshida S (2020). Utility of negative-pressure wound therapy for orocutaneous and pharyngocutaneous fistula following head and neck surgery. Head Neck.

[2] Benson EM, Hirata RM, Thompson CB (2015). Pharyngocutaneous fistula after total laryngectomy: a single-institution experience, 2001-2012. Am J Otolaryngol.

[3] Shah JP, Patel SG, Singh B (2023). Jatin Shah's Head and Neck Surgery and Oncology. Jatin Shah's Head and Neck Surgery and Oncology. Fourth ed. Philadelphia: Elsevier/Mosby.

[4] Steinbichler TB, Wolfram D, Runge A (2021). Modified vacuum-assisted closure (EndoVAC) therapy for treatment of pharyngocutaneous fistula: case series and a review of the literature. Head Neck.

[5] Paydarfar JA, Birkmeyer NJ (2006). Complications in head and neck surgery: a meta-analysis of postlaryngectomy pharyngocutaneous fistula. Arch Otolaryngol Head Neck Surg.

[6] Wang M, Xun Y, Wang K, Lu L, Yu A, Guan B, Yu C (2020). Risk factors of pharyngocutaneous fistula after total laryngectomy: a systematic review and meta-analysis. Eur Arch Otorhinolaryngol.

[7] Wieczorek A, Fijuth J, Michalski W (2002). The results of postoperative irradiation for locally advanced carcinoma of the larynx. J Oncol.

[8] Khoo MJ, Ooi AS (2021). Management of postreconstructive head and neck salivary fistulae: a review of current practices. J Plast Reconstr Aesthet Surg.

[9] Loaec E, Vaillant PY, Bonne L, Marianowski R (2014). Negative-pressure wound therapy for the treatment of pharyngocutaneous fistula. Eur Ann Otorhinolaryngol Head Neck Dis.

[10] Teixeira S, Costa J, Bartosch I, Correia B, Silva Á (2017). Management of pharyngocutaneous fistula with negative-pressure wound therapy. J Craniofac Surg.

[11] Marchese MR, Di Cesare T, De Corso E, Petracca M, Oliveto G, Almadori G (2022). Botulinum neurotoxin A in the treatment of pharyngocutaneous fistula after salvage surgery in head and neck cancer patients: our preliminary results. Curr Oncol.

[12] E Ferri, E Armato, D Fischetto, F Ianniello (2008). The role of botulinum toxin-A in the treatment of post-laryngectomy pharyngocutaneous fistula. Int J Otolaryngol.

